# Influence of simethicone and fasting on the quality of abdominal ultrasonography in New Zealand White rabbits

**DOI:** 10.1186/s13028-017-0316-x

**Published:** 2017-07-17

**Authors:** Kassy Gomes da Silva, Carla de Andrade, Cristina Santos Sotomaior

**Affiliations:** Graduate Program in Animal Science, School of Life Sciences, Pontifícia Universidade Católica do Paraná (PUCPR), Curitiba, Paraná 80215-901 Brazil

**Keywords:** Anti-foaming, Jejunum, *Oryctolagus cuniculus*, Rabbit, Simethicone, Ultrasonography

## Abstract

Presence of significant quantities of gas in the intestines may hinder a proper conduction of abdominal ultrasonography. In humans, preparatory techniques are used to solve this, but measures to avoid ultrasonographic complications due to intestinal gas in rabbits have not been reported. The objective of this study was to evaluate the influence of fasting and simethicone administered orally on the quality of ultrasonographic images of the gallbladder, kidneys, and jejunum in adult New Zealand White (NZW) rabbits. A total of 28 adult NZW rabbits were included in a crossover design study, involving four groups: F: fasting for 4–6 h before the examination; FS: fasting and application of simethicone (20 mg/kg, orally) 20 to 30 min before the examination; S: application of simethicone 20–30 min before the examination without fasting; and C: controls without fasting and no application of simethicone. Evaluation of the ultrasonographic images was done in terms of percentage of visualization of each organ and image quality using a 3-point scoring system (unacceptable, acceptable, or excellent). The kidneys and the gallbladder were visualized at an equal frequency in all groups, while the jejunum was visualized more frequently in the FS group. The image quality scores for gallbladder, right kidney, and left kidney was similar for all groups, but for the jejunum, a higher number of images with acceptable scores was found within the FS group.

## Findings

Ultrasonography has become a routine procedure for many veterinary practices, and recommendations for its use in rabbits have been published [1, 2] with multiple applications been reported recently [3, 4].

In rabbits, the stomach and intestines represent a significant portion of the abdominal cavity [5, 6]. The presence of significant quantities of gas in the intestines may hinder a proper conduction of abdominal ultrasonography [2, 7]. In humans, methods such as fasting, laxatives, anti-foaming agents, and water administered orally are used in an attempt to reduce the influence of gas on the quality of ultrasonographic imagining [8, 9]. Twelve hours of fasting before examination is recommended in humans, dogs and cats [10].

Simethicone is an anti-foaming agent comprised of a chemical mixture of polydimethylsiloxane and hydrated silica gel, and is used in rabbits to aid the degradation of gas bubbles associated with abdominal bloating [11]. The use of simethicone for ultrasound examinations is still a matter of debate [12], and animal studies have not been published. The aim of this study was to evaluate the influence of fasting and simethicone administered orally on the image quality obtained in abdominal ultrasonography in rabbits.

The study involved 28 adult New Zealand White rabbits (15 females, 13 males), with a mean weight of 4.37 ± 0.70 kg. It was a crossover design study, so every rabbit participated in all four groups, resulting in 112 examinations.

The groups were as follows: F: fasting for 4–6 h before the examination; FS: fasting and application of simethicone (20 mg/kg, orally) 20–30 min before the examination; S: application of simethicone 20–30 min before the examination without fasting; and C: controls without fasting and no application of simethicone. All animals had free access to water, feed, and hay; however, during fasting periods, feed was withheld. All rabbits were individually housed in suspended wire cages, with an automatic water dispenser and a manual feeder.

The fasting period was determined as described by Whittington [13]. A 20 mg/kg peroral dose of simethicone was used according to [14], and this was administered 20–30 min before the examination (adapted from [15]). A dorsal recumbent position on a foam V-trough pad was the standard position used during procedures. The hair-coat on the abdomen was clipped, and acoustic gel was applied to the abdominal area immediately preceding the beginning of the examination. A microconvex (4–8.0 MHz) transducer (Ecovet3, Chison, China) was used to perform all examinations.

The organs evaluated were gallbladder, right and left kidney, and the jejunum. The gallbladder was chosen because its direct relation to the digestive system and a segment of the jejunum because its easy standardization to localize it, which is medial and cranial to the left kidney [6]. Presence of gas in the bowel also produces obscure images of the right and left kidneys in the dorsal recumbency position; hence, both organs were included in the study. The duodenum can compromise visualization of the right kidney by either obscure the kidney or distort the image quality, while left kidney imaging can be compromised by the jejunum and colon [16].

A single veterinarian performed all abdominal ultrasounds and evaluated all images. All structures were evaluated using both transverse and longitudinal images. The overall gain and depth adjustments were set for each organ while performing the examination with the frequency at 8.0 MHz.

To assess the quality of imaging, two features were evaluated. First, it was assessed if the gallbladder, right and left kidney, and the jejunum could be visualized at all without regard to the image quality. Then the image quality was determined using a 3-point score adapted from [8] (1) “unacceptable,” whereby the image quality did not allow an adequate organ evaluation and a new examination was recommended; (2) “acceptable,” whereby the image quality was adequate for clinical purposes and there was no need to repeat the examination; and (3) “excellent,” whereby the image quality allowed for a clear definition of the anatomy of the organ.

Statistical analysis was performed using the Fisher’s Exact Test for the frequency of organ visualization, and the Kolmogorov–Smirnov test for comparing the distribution of image quality scores between groups. A 5% level of significance was set for both tests. Data were stored and analysed using Microsoft Excel 2010^®^.

The right and left kidneys were visualized in all rabbits of all groups. Gallbladder visualization ranged from 90 to 92%, with no significant difference between groups (Table 1). Visualization of the jejunum was equal for groups C, S, and F, but was more frequently visualized in FS group (P < 0.05) (Table 1).

**Table 1:** Frequency of visualization of the gallbladder and jejunum in adult NZW rabbits

Group	Gallbladder (%)	Jejunum (%)
C (n = 112)	90.2	86.6
S (n = 112)	90.2	87.5
F (n = 112)	92.0	87.5
FS (n = 112)	91.1	94.6*

*C* control, *S*: simethicone alone, *F* fasting alone, *FS* fasting and simethicone

* P < 0.05

**Fig. 1:**
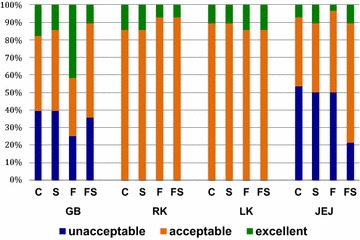
Image quality scores for gallbladder (GB), right kidney (RK), left kidney (LK) and jejunum (JEJ), compiled for each treatment in NZW adult rabbits. *C* control, *S* simethicone alone, *F* fasting alone, *FS* fasting and simethicone

The image quality scores for the gallbladder and kidneys were similar for all groups. However, for the jejunum, the image quality was significantly better for the FS group than for the other groups (P < 0.05). The FS group also had a lower frequency of images considered “unacceptable” than to other groups (Fig. 1).

In humans, fasting promotes dilatation of the gallbladder, thus favouring its viewing during sonographic examinations [17]. In the current study, dilatation of the gallbladder was not observed, which is possibly due to the relatively large amount of bile continuously secreted by rabbits [18]. Moreover, hormones such as secretin and gastrin that normally regulate the secretion of bile in other species such as dogs and cats may not be as effective in rabbits [19]. The fasting period used in this study (4–6 h) was probably not sufficient to affect the level of bile secretion and it should also be noted that despite the fasting period, rabbits consume cecotrophes [8], the intake of which was not prevented during this study.

The FS group showed a higher percentage of “acceptable” images of the jejunum than the other groups, indicating that the quality of ultrasound imaging in these animals was improved by a combination of fasting and simethicone administration. The length of fasting period used was based on the maximum fasting period suggested for rabbits [13], while avoiding gastrointestinal disorders. Fasting alone, as a method of preparation for abdominal ultrasound in rabbits, did not show better results than the controls as also shown for humans [8, 20] and dogs [21]. The combination of fasting and simethicone proved suitable to obtain mostly “acceptable” and “excellent” scores for images of the jejunum. In rabbits, simethicone is indicated in the treatment of abdominal discomfort caused by gas [11]. The recommended dose for this purpose ranges from 20 to 130 mg/kg; in this study, it was chosen 20 mg/kg because it was the lowest one [13–15]. The pre-treatment period of 20–30 min was adapted from the recommended 1 h for treatment of bowel distension in rabbits caused by gas [14]. Moreover, a shorter period between administration and ultrasonography allows the sonographer to administer the simethicone rather than the owner.

In conclusion, fasting or simethicone administration alone did not influence the image quality for the kidneys and gallbladder in adult New Zealand White rabbits. For imaging of the jejunum, there was improvement in visualization and image quality following 4–6 h of fasting in combination with 20 mg/kg of oral simethicone. This preparatory method is recommended for abdominal ultrasonography in rabbits.
